# Improving Clinician's Knowledge and Comfort with Prenatal and Postpartum Employment Laws: A Pilot Intervention

**DOI:** 10.1089/whr.2022.0053

**Published:** 2022-11-11

**Authors:** Shauntell Luke, Gabriel Carrillo, Hannah Demeritt, Tracy Truong, Melody Baldwin, Richalle Sullivan, Katrina Avery, Geeta Swamy, Sarahn Wheeler

**Affiliations:** ^1^ Department of Obstetrics and Gynecology, Duke University School of Medicine, Durham, North Carolina, USA.; ^2^ Health Justice Clinic, Duke Law School, Durham, North Carolina, USA.; ^3^ Department of Biostatistics and Bioinformatics, Duke University, Durham, North Carolina, USA.

**Keywords:** employment, prenatal, postpartum, laws, pregnancy

## Abstract

**Background:**

It is common for pregnant people in the United States to continue to work throughout their pregnancy. Pregnant people may need leave time or other accommodations to continue working safely. It is imperative that obstetric providers are knowledgeable regarding laws that govern the prenatal and postpartum period to provide appropriate counseling and medical documentation in support of requests for leave time and workplace accommodations.

**Methods:**

We created a virtual training for obstetric clinicians regarding employment considerations in the prenatal and postpartum period. The training details the federal laws that govern this period, when and how to request reasonable accommodations from an employer, and provides resources for clinicians to use when they believe pregnancy-related discrimination has occurred. We conducted pretest and post-test surveys to assess change in knowledge about employment laws and comfort with counseling patients.

**Results:**

There were 61 clinicians who completed the training (50.4% response rate). The majority (88%, *n* = 54) of respondents reported no prior formal training about employment laws in pregnancy. On the pretraining self-assessment, >93% (*n* = 57) of participants felt they had minimal or very minimal knowledge regarding lactation and pregnancy-related accommodations, compared with >91% (*n* = 55) feeling very or somewhat knowledgeable after the training. The mean percent correct on the knowledge assessment increased from 55% to 67% on the pre- and post-test knowledge questions, respectively.

**Discussion:**

Our findings suggest an on-demand virtual training can improve knowledge and comfort for obstetric clinicians about federal employment laws in pregnancy and postpartum.

## Introduction

In the United States, >50% of pregnant people work full time during pregnancy.^1,2^ Approximately 80% of patients pregnant with their first child continue working through the month of their due date.^1,3,4^ In addition, ∼41% of pregnant patients are the primary or sole financial provider for their family.^2,5^ Although it is common to work during pregnancy, the United States does not have a nationally funded paid parental leave program.^1,3,6^ Despite the absence of a federal parental leave policy, there are limited unpaid job protections provided by federal laws, including the Family Medical Leave Act (FMLA), Pregnancy Discrimination Act (PDA), and Americans with Disabilities Act (ADA).

Furthermore, there are also some state-specific statutes that govern workplace accommodations for pregnant and postpartum patients. These laws are complex and nuanced, including whether or not the patient is deemed to have a “complicated” or “uncomplicated” pregnancy, which influences the type of workplace accommodations that a patient is entitled to receive.^1,6^

Obstetric clinicians are an important resource for patients as they navigate parental leave, job duty modifications or other accommodations during pregnancy and the postpartum period. Although clinicians are an important resource, they often have limited formal training regarding federal and state labor laws that impact patients during pregnancy and postpartum. The objective of this study was to pilot an on-demand virtual training program about federal and state laws that govern employment leave, job duty modifications, or other accommodations during pregnancy and postpartum (referred to as employment considerations throughout the article) in North Carolina. We hypothesized the training program will increase knowledge and comfort with counseling patients about employment considerations during pregnancy and postpartum.

## Materials and Methods

We conducted a pilot study of an online training with multidisciplinary members of the obstetric clinical care teams at a single institution.

### Eligibility criteria and recruitment procedures

The Duke Health Institutional Review Board (Pro00105018) approved the study. We partnered with six obstetrical clinics within the Duke Health system. All obstetric clinical providers, including physicians, advanced practice practitioners, residents, and fellows, were invited to complete the training. In addition, we engaged the medical directors of each clinic to identify nurses, medical assistants, social workers, and clerical staff who participate in processing employment documentation requests. Finally, we recruited 4th-year medical students who successfully matched into Obstetrics and Gynecology (OB/GYN) residency to participate in the pilot study. Potential participants were recruited *via* e-mail solicitation.

### Data collection and analysis

Study data were collected and managed using Research Electronic Data Capture (REDCap™),^7^ a secure web-based application designed to support data capture for research studies. Participants were consented electronically before initiating any data collection. Participants had a minimum of ∼2 months and a maximum of 6 months to complete the training.

### Pretest comfort and self-assessed knowledge quiz

The training began with a five-question comfort pretest survey that assessed the participant's comfort level with counseling and self-perceived knowledge regarding the laws governing the pregnancy and postpartum period. The answer choices were on a 5-point Likert scale ranging from “very,” “somewhat,” “minimal,” and “very minimal comfort.”

### Pretest knowledge quiz

The comfort pretest survey was followed by 10 pretest knowledge questions with true/false/unsure and multiple-choice answer choices. This quiz aimed to objectively assess the participant's knowledge regarding the laws governing the prenatal and postpartum period.

After completion of the comfort survey and knowledge questionnaires, participants received access to the training.

### Training modules

The content for the modules was developed by a multidisciplinary team of physicians and health justice lawyers. The training included seven online modules narrated by an MD candidate and an MD/JD candidate. Participants were able to start and stop the training as needed. The modules covered a range of topics including the FMLA, Affordable Care Act, ADA, PDA, how to request reasonable accommodations, and resources such as a work note template (Supplementary Appendix SA1 and SA2). The individual modules were between 2 and 8 minutes in length (Table 1).

**Table 1.** Brief Summary of Training Modules

Module	Summary	Length (minutes:seconds)
1. Introduction	Brief introduction to the training, including why it is important and why it was created	3:11
2. Pregnancy and work	Discussion about federal laws that govern employment in the prenatal and postpartum period (Appendix) • Title VII of the Civil Rights • PDA • FMLA • ADA • ACA “Break Time for Mothers”	3:40
3. Workplace accommodations	How to suggest accommodations based on classification of “complicated” vs. uncomplicated pregnancy and the nature of the patient's job	8:21
4. Effective work note	Best practices for writing a work note to an employer including a downloadable work template	4:13
5. Leave policies	Understanding the role of FMLA and how it governs leave, what paperwork to complete and who qualifies for this	3:21
6. Lactation accommodations	Understanding the role of ACA and how it governs lactation, who is eligible and how providers can help secure this for patients	2:40
7. Medical-legal advocacy	Resources on how to advocate and who to contact	1:32

ACA, Affordable Care Act; ADA, Americans with Disabilities Act; FMLA, Family Medical Leave Act; PDA, Pregnancy Discrimination Act.

### Post-training surveys

After the training, participants completed post-test comfort and knowledge surveys. These surveys included similar questions to the pretest surveys. Participants were also asked about their experience with the training (*e.g.*, the length and relevance of the training). Finally, participants were asked to complete a demographic survey, which included race, ethnicity, obstetric clinic location, professional degree, and length of time working within their respective field.

Combined with pretest and post-test surveys, we estimated the entire training was ∼30 minutes.

### Analysis

The primary outcomes were change in knowledge (post-test quiz score minus pretest quiz score) and self-assessment (self-assessed comfort/knowledge survey post-test score minus pretest score). Wilcoxon signed rank test was used to compare pre- and post-training knowledge quiz scores and self-assessment survey responses. Descriptive statistics were presented for demographics.

## Results

### Demographics

We invited 121 individuals to participate in our study, the majority of whom were physicians (*n* = 77) and 44 individuals with varying clinical roles (Table 2). The completion rate of the training was 50.4% (61 respondents out of 121 invitees) overall and ranged from 89% among medical students to 38% among staff members (including certified medical assistants and clinical social workers). The majority (*n* = 59, 97%) reported they had no prior formal training related to employment law relevant to pregnant/postpartum patients. Only 34% (*n* = 21) of the cohort stated they often or very often counsel their patients on employment considerations.

**Table 2.** Demographics and Response Rate

Degree/position	Total invitees (***N*** = 121)	Respondents (***N*** = 61)	Response rate (50.7%), %
Physicians	77	36	46
Advanced practice providers	10	5	50
Nurses	12	6	50
Staff (certified medical assistants and clinical social workers)	13	5	38
Medical student	9	8	89
Prefer not to answer	—	1	N/A

N/A, not applicable.

### Change in knowledge

The median percent correct on the 10-question knowledge quiz increased from 60% before the training to 70% after training completion (*p* < 0.001; Table 3). Comparing pre- and post-test question answers, the number of participants who selected the correct answer increased for all 10 of the quiz questions. On the pretest, there were only two questions that 75% of the respondents answered correctly. On the post-test assessment there were only three questions that 75% or less of participants answered incorrectly. On the other seven questions, >90% of respondents answered correctly on the post-test.

**Table 3.** Pre- and Post-Training Knowledge

Question	Percentage of respondents correct	
	Pretest	Post-test
1. In writing an effective work note, it is preferred to only suggest a work restriction or modification when medically necessary. Answer choices: true, false, or not sure		
Correct answer (true)	41	93
2. In recent estimates, approximately what percentage of nulliparous pregnant women worked within 1 month of their due date? Answer choices: (A) 20%, (B) 60%, (C) 80%, or (D) 95%		
Correct answer (C)	56	95
3. A pregnant mother whose employment requires standing for more than 10 or more hours recently complained of lower back pain. She continues to have this pain despite conservative management. What would be your suggestion? Answer choices: (A) Decrease her shifts to 6 hours, reducing her overall pay, (B) Provide a floor mat that helps to alleviate back pressure or (C) switch to another position at the job that does not require standing but earns $2 less per hour than her current position		
Correct answer (B)	70	90
4. According to the American Committee for Obstetrics and Gynecology, how would you counsel a patient about lifting restrictions at 32 weeks gestation? Answer choices: (A) She can only lift 5 lbs twice a day, (B) She can lift up to 50 lbs up to 5 times a day from a maximum height of 3 feet, (C) Depends on factors such as how often and from what height the patient is lifting, (D) No restrictions exist		
Correct answer (C)	63	75
5. The United States is the only developed country that does not have a national paid maternity leave. Answer choices: true, false, not sure		
Correct answer (true)	80	97
6. The FMLA provides the benefits of 12 weeks of unpaid, job protected leave and continued health benefits for prenatal care appointments, birth, and care of a newborn. Answer choices: true, false, not sure		
Correct answer (true)	84	95
7. In order for your patient to receive FMLA benefits, the patient must be employed for 12 or more months, have worked at least 1250 hours, and the company must employ 50 or more people. Answer choices: true, false, not sure		
Correct answer (true)	69	97
8. The PDA provides accommodations for pregnant workers that are similar to other employees with temporary disability. Answer choices: true, false, not sure		
Correct answer (false)	7	5
9. Some salaried and most hourly employees are eligible for lactation accommodations provided by the ACA. Answer choices: true, false, not sure		
Correct answer (true)	74	97
10. The ADA requires that employers provide reasonable accommodations for pregnancy-related impairments assuming these accommodations do not cause undue hardship to the employer. Answer choices: true, false, not sure		
Correct answer (false)	3	8
**Overall median percent correct (*p* < 0.001)**	**60 [50, 60]**	**70 [60, 70]**

Values are in bold to indicate the emphasis.

^*^ *p*-Value from Wilcoxon signed rank test.

### Change in self-assessment

Before the training, self-assessed knowledge about lactation and pregnancy-related accommodations was evenly split between somewhat knowledgeable, minimal knowledge, and very minimal knowledge (31.1% [*n* = 19], 31.1% [*n* = 19], and 34.4% [*n* = 21], respectively). After the training, self-assessed knowledge increased with the majority of participants feeling very or somewhat knowledgeable (27.9% [*n* = 17], and 65.6% [*n* = 40], respectively; Table 4).

**Table 4.** Self-Assessment Pre- and Post-Training

	Pretest (***N*** = 61), ***n*** (%)	Post-test (***N*** = 61), ***n*** (%)
Self-assessed knowledge		
1. How knowledgeable do you feel about the federal and state law that govern lactation and pregnancy-related accommodations?		
Very minimal knowledge	21 (34.4)	1 (1.6)
Minimal knowledge	19 (31.1)	3 (4.9)
Somewhat knowledgeable	19 (31.1)	40 (65.6)
Very knowledgeable	2 (3.3)	17 (27.9)
2. How knowledgeable do you feel about the federal and state laws that govern pregnancy-related discrimination?		
Very minimal knowledge	23 (37.7)	1 (1.6)
Minimal knowledge	21 (34.4)	4 (6.6)
Somewhat knowledgeable	15 (24.6)	41 (67.2)
Very knowledgeable	2 (3.3)	15 (24.6)
Self-assessed comfort		
3. If you believed your patient was a victim of pregnancy-related discrimination, how comfort are you counseling the patient about their options?		
Very minimal comfort	25 (41.0)	2 (3.3)
Minimal comfort	24 (39.3)	10 (16.4)
Somewhat comfortable	9 (14.8)	34 (55.7)
Very comfortable	3 (4.9)	15 (24.6)
4. How often do you (will you) counsel your patients about employment considerations during their pregnancy?		
Never	3 (4.9)	1 (1.6)
Rarely	16 (26.2)	1 (1.6)
Sometimes	21 (34.4)	16 (26.2)
Often	16 (26.2)	29 (47.5)
Very often	5 (8.2)	14 (23.0)

A similar trend was observed regarding self-assessed knowledge about pregnancy-related employment discrimination. Before the training, the most common responses were very minimal (*n* = 23, 37.7%) or minimal knowledge (*n* = 21, 34.4%). After the training, there were 24.6% [*n* = 15], 34.4% [*n* = 21], and 37.7% [*n* = 23] of participants feeling somewhat knowledgeable, having minimal knowledge, and very minimal knowledge, respectively (Table 4).

Participants were also asked about their comfort counseling a patient experiencing pregnancy-related employment discrimination. Before the training, majority of respondents felt minimal or very minimal comfort (39.4% [*n* = 24] and 41.0% [*n* = 25], respectively). After the training, majority of respondents felt very and somewhat comfortable (24.6% [*n* = 15], 55.7% [*n* = 34]) on their ability to provide appropriate counseling (Table 4).

Participants were then assessed on how often they counseled patients about employment considerations during pregnancy before the training. Again, there was an almost even split with 26.2% [*n* = 16], 34.4% [*n* = 21] and 26.2% [*n* = 16] selecting they often, sometimes, and rarely counsel their patients on this topic. After training, they were asked how often they will counsel their patients in the future. We observed that 23.0% [*n* = 14], 47.5% [*n* = 29], and 26.2% [*n* = 16] plan to counsel their patients on employment considerations very often, often, and sometimes, respectively (Table 4).

## Discussion

In our pilot study of an on-demand virtual training about employment considerations in pregnancy and postpartum, obstetrical clinicians felt more comfortable and demonstrated increased knowledge after the training. This study adds to a very sparse body of literature examining obstetrical clinician's knowledge about employment considerations in the prenatal and postpartum period.

The majority of respondents in our study reported no prior training on employment laws and pregnancy. The lack of formal training is unlikely related to disinterest, as evident by the relatively high participation rate, and more likely related to time constraints during medical training and the demands of clinical practice. In addition, obstetrical training programs and clinical practices may lack the legal expertise needed to develop and disseminate concise, practical summaries of the key federal and state laws.

The complexity, rapidly evolving details, and variability of the laws necessitate collaboration with legal experts to provide relevant and accurate summaries of the salient legal points.^2^ Our study demonstrates a feasible pedagogy to deliver information about relevant laws that pertain to pregnancy and postpartum. Furthermore, the virtual, on-demand model for training can be used for refresher or updated trainings that may be needed due to dynamic changes in the law.

The literature demonstrates the potentially devastating impact of obstetrical clinicians being ill-informed about the legal aspects of pregnancy.^2,6,8^ For example, without knowledge about legal considerations for workplace accommodations, providers may unintentionally write ineffective work notes leading to a denial of any accommodations and/or loss of employment.^2,6,8^ Our training addresses this issue by educating providers on how to write an effective work note and by providing a template work note to support providers' role in encouraging safe employment parameters for our patients. In addition to training clinicians to provide effective documentation, the medical–legal advocacy portion of the training highlights the importance of initiating a conversation with patients about employment to gauge if reasonable accommodations are needed.^2^

Franco et al found more than half of the pregnant workers in their cohort felt they needed accommodations, yet 40% of this population never requested them.^9^ Without necessary, and in some cases legally protected, accommodations patients may be at increased risk for poor obstetrical outcomes. For example, a study examining the role of stress during pregnancy found increased levels of preterm birth were associated with a higher occupational fatigue score.^10^ Providing a work note detailing reasonable accommodations may be helpful in decreasing stress related to working during pregnancy.

Black and Hispanic people are more likely to work in a low-wage job, which may create additional challenges navigating employment during pregnancy and the postpartum.^5,11,12^ Based on the U.S. Census Bureau-administered American Community Survey, 20.9% of all pregnant workers are employed in low-wage jobs (earning $11.50 per hour or less); however, Black and Hispanic pregnant works are disproportionally represented in these jobs (30.0% and 31.3%, respectively).^12^

Pregnant workers employed in low-wage jobs are more likely to have physically demanding duties, for example, standing or lifting that may require modifications to ensure safety during pregnancy.^12^ Furthermore, these low-wage jobs are often associated with an inflexible work culture^3^; and >40% of full-time low-wage workers report limited ability to choose break times and ∼50% report little or no control over their work schedule.^13^ Our study demonstrates that an online training can empower clinicians with skills and tools that may be uniquely important for Black and Hispanic patients.

There are several strengths of our study. The training was developed *via* a multidisciplinary team, including obstetrical clinicians and lawyers (one MD, two JDs, a JD/MD candidate, two JD candidates, and one MD candidate) with expertise in employment law. The virtual on-demand training delivery was tailored to an obstetrical clinician's busy schedule. Furthermore, our pilot includes all clinical team members who participate in prenatal and postpartum employment counseling/documentation, including physicians, advance practicing providers, nurses, medical assistants, and clerical staff. Finally, although participants were not compensated, there was a 50% response rate, and >85% respondents felt the training was relevant to their clinical practice.

Despite the importance of our pilot study, limitations must be considered. First, our results may be subject to selection bias because the individuals who elected to participate in the training are more likely to be interested in the topic compared with those who declined. The study is also limited by a relatively short length of the knowledge survey (10 questions). We included only 10 questions to accommodate the busy schedules of the participants; however, the brief nature of the survey may obscure subtle changes in knowledge. Furthermore, the assessment of knowledge immediately follows the training, and this study does not assess durable changes in participant knowledge.

Despite these limitations, our findings suggest meaningful clinically relevant training can be provided for obstetric clinicians using a virtual format. Future directions of this research will include training providers at multiple health care systems (academic and nonacademic), and evaluating the impact of the training on provider practice, including quality of work notes, accuracy of FMLA paperwork, and frequency of patient counseling about employment in pregnancy.

## Conclusion

Currently, there are important policy debates about whether and how to offer paid leave and other employment accommodations during pregnancy and postpartum in the United States. Obstetrical clinicians will need tools and resources to stay up to date as policy progresses. Increased training on the laws and the limitations of legal protections can equip clinicians to be more effective advocates to ensure their patients receive all the current and any future protections offered by the law.

## Supplementary Material

Supplemental data

Supplemental data

## Supplementary Material

Supplementary Appendix SA1

Supplementary Appendix SA2

## Abbreviations Used

ACA: Affordable Care Act
ADA: Americans with Disabilities Act
FMLA: Family Medical Leave Act
NCATS: National Center for Advancing Translational Sciences
NIH: National Institutes of Health
OB/GYN: Obstetrics and Gynecology
PDA: Pregnancy Discrimination Act
