# Supplementary material for: Improving Clinician's Knowledge and Comfort with Prenatal and Postpartum Employment Laws: A Pilot Intervention
Source: Womens Health Rep (New Rochelle). 2022 Nov 11;3(1):924–30. doi: 10.1089/whr.2022.0053 (PMC9712045; doi:10.1089/whr.2022.0053)
Supplement: Supplemental data [file Suppl_AppendixSA1.docx]

| **Appendix A. Employment Laws that Govern the Prenatal and Postpartum Period** | | |
| --- | --- | --- |
| Employment Laws | Summary of Law | Limitations |
| Title VII of the Civil Rights | Prohibits discrimination in employment based on sex, religion, race, color, national origin and sexual orientation | Did not specifically include disabled and pregnant women |
| Pregnancy  Discrimination Act | Prohibits discrimination based on pregnancy in employment i.e. health insurance, job assignments, leave, hiring, promotions, etc. | Accommodations are made as long as they do not cause undue hardship to the employer, must be employed at a company with at least 15 employees |
| Family Medical Leave Act (FMLA) | Up to 12 weeks of unpaid leave if an employee if they work for an eligible employer* AND has worked for has been employed for at least 12 months, worked 1250 within 12 months, works for  employer that has at least 50 employees    *Eligible employer: private sector company with at least 50 employees, public agency or public or private elementary or secondary school | Employees who do not meet these criteria are not eligible |
| Americans with Disabilities Act | Employers must provide reasonable accommodations to pregnant mothers with temporary disability | Must be employed at a company with at least 15 employees |
| Affordable Care Act,  “Break Time for  Nursing Mothers” | Nursing mothers are allowed to pump at work for up to 12 months after delivery and companies must provide appropriate breaks and storage for breast milk | Most but not all hourly and salaried employees are covered |
