# Supplementary material for: Improving Clinician's Knowledge and Comfort with Prenatal and Postpartum Employment Laws: A Pilot Intervention
Source: Womens Health Rep (New Rochelle). 2022 Nov 11;3(1):924–30. doi: 10.1089/whr.2022.0053 (PMC9712045; doi:10.1089/whr.2022.0053)
Supplement: Supplemental data [file Suppl_AppendixSA2.docx]

| **Appendix B. Example Work Note Template for Employers** |
| --- |
| [Clinic Letterhead]  [Date]  To Whom It May Concern:  I saw [patient name] in clinic today. In my medical opinion, [patient name] is able to work with reasonable accommodations. While standing for prolonged periods of time is not strictly prohibited in pregnancy, we generally recommend standing for no more the two hours without a break. Due to her pregnancy-related medical condition, please consider providing a sitting stool to avoid disruptions to [patient name’s] work.  Thank you for your partnership in a creating safe environment for pregnant workers.  Please do not hesitate to contact our office if you have additional questions.  Sincerely,  Clinician Signature  Credentials |
